# MINDFULNESS ENHANCES PROACTIVE BENEFITS OF REAPPRAISAL USE FOR YOUNGER BUT NOT OLDER ADULTS

**DOI:** 10.1093/geroni/igad104.3472

**Published:** 2023-12-21

**Authors:** Bruna Martins-Klein, Ziyuan Chen, Mabel Shanahan, Gregory Theos, Kristin Heideman, Irina Orlovsky, Clara DeFontes, Elizabeth Alwan

**Affiliations:** University of Southern California, Los Angeles, California, United States; University of Southern California, Los Angeles, California, United States; Amherst College, Amherst, Massachusetts, United States; Advanced Science & Math Academy Charter School, Marlborough, Massachusetts, United States; Yale University, New Haven, Connecticut, United States; University of Massachusetts Amherst, Amherst, Massachusetts, United States; University of Massachusetts Amherst, Amherst, Massachusetts, United States; National Academy of Public Administration, Washington, District of Columbia, United States

## Abstract

Reappraisal and mindfulness are associated with enhanced mood and emotion regulation. Cognitive control is key to both processes, and despite age-related declines in cognitive control, older adults report greater use of both compared to younger adults. The Dual mechanisms of control (DMC) framework posits that cognitive control can be engaged, either in anticipation of a task (proactive control) to enhance task performance, or in-the-moment (reactive control). In classic cognitive tasks, older adults show limited benefits from proactive control and intact reactive control, while mindful individuals show similar performance using both control modes. However, the role of trait mindfulness on proactive versus reactive control of reappraisal has never been explored across the lifespan. In this study, 41 younger (18-29) and 40 older adults (60-85) were cued to reappraise or view 80 negative images following a 4-s proactive or a 500-ms reactive delay, tracking pupil response as a measure of physiological arousal and effort. Multilevel models revealed that mindfulness was associated with greater proactive reappraisal benefits in younger adults. In contrast, mindfulness was associated with diminishing benefits of proactive versus reactive reappraisal in older adults; for low mindfulness, proactive benefits were found, and for high mindfulness, reactive control benefitted reappraisal more than proactive control (p < .001). For image viewing, proactive control benefitted older but not younger adults. Findings highlight the importance of considering the interaction of mindfulness and age in emotion regulation, and highlight the need of optimizing the temporal dynamics of reappraisal-based strategies in mental health interventions across the lifespan.

